# Supplementary material for: Directed evolution of AAV accounting for long-term and enhanced transduction of cardiovascular endothelial cells in vivo
Source: Mol Ther Methods Clin Dev. 2021 Jun 4;22:148–61. doi: 10.1016/j.omtm.2021.05.015 (PMC8397840; doi:10.1016/j.omtm.2021.05.015)
Supplement: Document S1. Materials and methods and Figures S1–S3 [file mmc1.pdf]

OMTM, Volume *22*

Supplemental information

Directed evolution of AAV accounting for long-term and enhanced transduction of cardiovascular endothelial cells *in vivo*

Y.B. Liu, B.C. Xu, Y.T. Chen, X. Yuan, J.Y. Liu, T. Liu, G.Z. Du, W. Jiang, Y. Yang, Y. Zhu, L.J. Chen, B.S. Ding, Y.Q. Wei, and L. Yang

MATERIALS AND METHODS

Co-localization of AAV-mediated transgene expression and cardiac endothelial cells

3×10^{11} vector genomes of EC71-CMV-GFP vector was injected into male 7-week-old C57BL/6J mice via tail vein. After two weeks, the heart was collected and fixed with 4% paraformaldehyde at 4°C for 12 hours, and then transferred to 30% sucrose for dehydration at 4°C for 24 hours. Tissue for immunostaining was embedded in OCT and 9 μm frozen sections were prepared. Sections were fixed with 4% paraformaldehyde at room temperature for 5 minutes and incubated overnight at 4°C with the rat anti-CD31 (BD Pharmingen, 557355) and chicken anti-GFP (Abcam, ab13970) primary antibodies at a 1:200 dilution in blocking buffer (1% BSA, 10% donkey serum and 0.025% Tween 20 in PBS). Sections were later protected from light and incubated for one hour at room temperature with 1% BSA and 5% donkey serum in PBS containing both donkey anti-rat IgG antibodies conjugated with Alexa Fluor 647 (Jackson, 712-605-150) and donkey anti-chicken IgG, Alexa Fluor 488 (Jackson, 703-545-155) secondary antibodies at a 1:500 dilution. The images were visualized with a confocal microscope (Zeiss) and analyzed with ZEN software. Images were not manipulated in any way except to make brightness and contrast adjustments.

Characterization of Flt1 and eNOS promoter

The AAV-eNOS-luc plasmid was constructed by replacing the CMV promoter in AAV-CMV-luc plasmid with the eNOS promoter[1] (-1033/+22) from the pGL2 enhancer-F1 LUC plasmid (Addgene). EC71-Flt1-luc and EC71-eNOS-luc vectors were produced using the three-plasmid co-transfection method and CsCl gradient density centrifugation. 3×10^{11} or 1×10^{12} vector genomes of these vectors were injected into the male 7-week-old C57BL/6J mice via tail vein. Three weeks later, mouse heart, liver, brain, and lung tissues were harvested for luciferase assay as previously described.

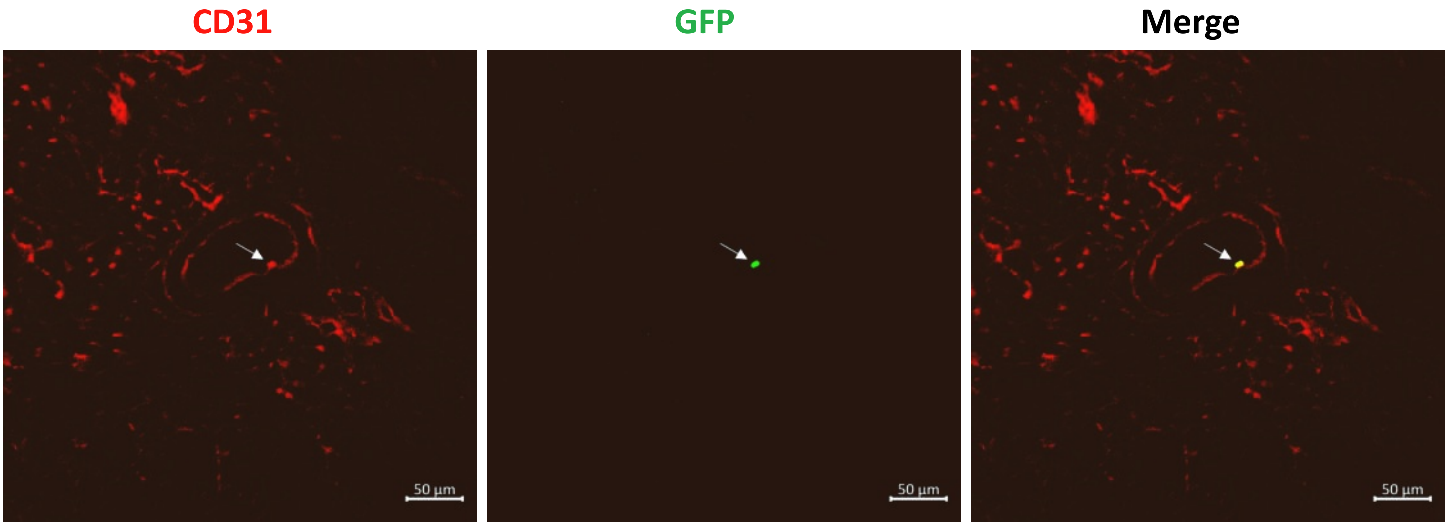

Figure S1. EC71 transduced cardiac endothelial cells in vivo. 3x10^{11} vector genomes of EC71-CMV-GFP were injected into the young adult C57B6 mice intravenously and transgene expression within mouse heart was detected by immunostaining two weeks later. The representative images for GFP transgene expression (green) in the cardiac endothelial cells co-stained with CD31 marker (red) were demonstrated. The white arrows showed co-staining.

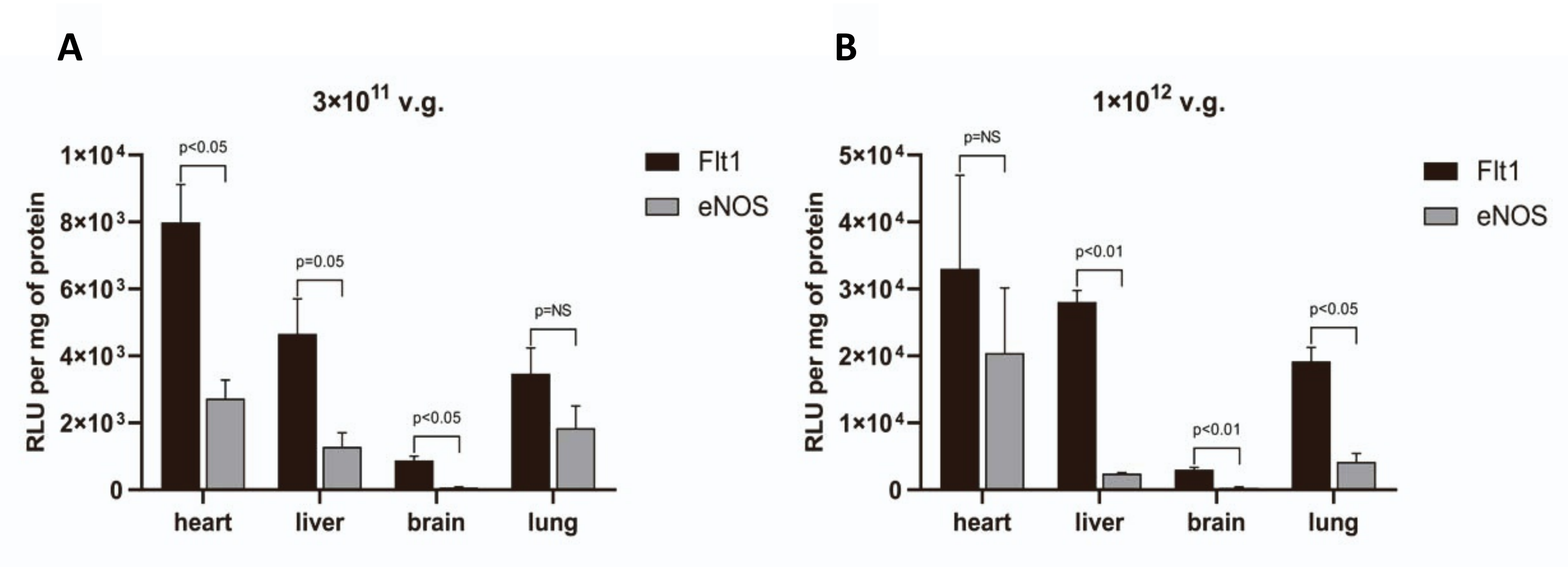

Figure S2. Characterization of the endothelial-specific Flt-1 and eNOS promoters. 3×10^{11} (A) or 1×10^{12} (B) vector genomes of EC71 vector encompassing luciferase gene were injected intravenously into adult C57B6 mice. Three weeks later, luciferase activities among mouse tissues were measured to compare these two promoters on their strength and tissue specificity. Data are mean values ± SD.

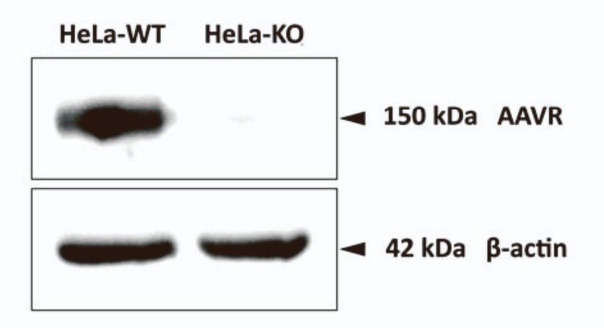

Figure S3. AAVR expression in HeLa cell strains. HeLa-WT and HeLa-KO cell lysates were separated by SDS-PAGE and immunoblotted with a commercial AAVR antibody. One approximate 150 kDa band correspondent to glycosylated AAVR was observed for the HeLa-WT cells but lacked for HeLa-KO cells.

REFERENCES

1. Zhang, R., Min, W., and Sessa, W. C. (1995). Functional analysis of the human endothelial nitric oxide synthase promoter. Sp1 and GATA factors are necessary for basal transcription in endothelial cells. J. Biol. Chem. 270, 15320-15326.
