# Supplementary material for: Directed evolution of AAV accounting for long-term and enhanced transduction of cardiovascular endothelial cells in vivo
Source: Mol Ther Methods Clin Dev. 2021 Jun 4;22:148–61. doi: 10.1016/j.omtm.2021.05.015 (PMC8397840; doi:10.1016/j.omtm.2021.05.015)
Supplement: Document S2. Article plus supplemental information [file mmc2.pdf]

Molecular Therapy
Methods & Clinical Development
Original Article

Directed evolution of AAV accounting for long-term and enhanced transduction of cardiovascular endothelial cells *in vivo*

Y.B. Liu,[1] B.C. Xu,[2] Y.T. Chen,[3] X. Yuan,[2] J.Y. Liu,[4] T. Liu,[5] G.Z. Du,[5] W. Jiang,[6] Y. Yang,[7] Y. Zhu,[8] L.J. Chen,[4] B.S. Ding,[3] Y.Q. Wei,[2] and L. Yang[1]

[1]Department of Cardiology and Laboratory of Gene Therapy for Heart Diseases, State Key Laboratory of Biotherapy, West China Hospital, Sichuan University and Collaborative Innovation Center for Biotherapy, Chengdu, Sichuan, China; [2]Laboratory of Aging Research and Cancer Drug Target, State Key Laboratory of Biotherapy and Cancer Center, National Clinical Research Center for Geriatrics, West China Hospital, Sichuan University, Chengdu, Sichuan, China; [3]Key Laboratory of Birth Defects and Related Diseases of Women and Children of MOE, State Key Laboratory of Biotherapy, West China Second University Hospital, Sichuan University and Collaborative Innovation Center for Biotherapy, Chengdu, Sichuan, China; [4]State Key Laboratory of Biotherapy and Cancer Center, West China Hospital of Sichuan University, Chengdu, China; [5]Department of Anesthesiology, Translational Neuroscience Center, West China Hospital, Sichuan University, Chengdu, Sichuan, China; [6]Molecular Medicine Research Center, State Key Laboratory of Biotherapy, West China Hospital, Sichuan University, Chengdu, Sichuan, China; [7]State Key Laboratory of Biotherapy and Cancer Center, West China Hospital, Sichuan University and Collaborative Innovation Center, Chengdu, Sichuan, China; [8]Department of Cardiology, West China Hospital, Sichuan University, Chengdu, Sichuan, China

Cardiac endothelial cells (ECs) are important targets for cardiovascular gene therapy. However, the approach of stably transducing ECs *in vivo* using different vectors, including adeno-associated virus (AAV), remains unexamined. Regarding this unmet need, two AAV libraries from DNA shuffling and random peptide display were simultaneously screened in a transgenic mouse model. Cardiac ECs were isolated by cell sorting for salvage of EC-targeting AAV. Two AAV variants, i.e., EC71 and EC73, enriched in cardiac EC, were further characterized for their tissue tropism. Both of them demonstrated remarkably enhanced transduction of cardiac ECs and reduced infection of liver ECs in comparison to natural AAVs after intravenous injection. Significantly, persistent transgene expression was maintained in mouse cardiac ECs *in vivo* for at least 4 months. The EC71 vector was selected for delivery of the endothelial nitric oxide synthase (eNOS) gene into cardiac ECs in a mouse model of myocardial infarction. Enhanced eNOS activity was observed in the mouse heart and lung, which was correlated with partially improved cardiac function. Taken together, two AAV capsids were evolved with more efficient transduction in cardiovascular endothelium *in vivo*, but their endothelial tropism might need to be further optimized for practical application to cardiac gene therapy.

INTRODUCTION

The vascular endothelium plays a pivotal role in the regulation of vascular function and homeostasis and is also an attractive therapeutic target for cardiovascular diseases.[1] Adenovirus can efficiently transduce endothelial cells (ECs) in the 30% to 50% range. Furthermore, adenovirus-mediated gene transfer has been successfully applied in the treatment of a variety of cardiovascular diseases.[2,3] However, the application of adenoviral vector often involves induction of the immune response and rapid loss of transgene expression, rendering assessment of the long-term efficacy of endothelium-targeting gene therapy impossible, which hinders efficient treatment of chronic cardiovascular diseases by this approach.

Adeno-associated virus (AAV) is a promising vector for *in vivo* gene therapy with respect to its higher safety profile and mediated stable transduction in postmitotic cells. Two gene therapy drugs, Luxturna and Zolgensma, have been approved based on AAV vectors by the US Food and Drug Administration for the treatment of inherited retinal disease and spinal muscular atrophy, respectively. However, ECs were relatively resistant to transduction by natural AAV serotypes, especially *in vivo*. For instance, low transduction of AAV2 in both human umbilical vein ECs (HUVECs) and rabbit carotid artery ECs was thought to result from massive deposits of its glycan receptor, heparin sulfate proteoglycan, in the extracellular matrix.[4] In another study, AAV1 and AAV5 showed higher transduction of human and rat aortic ECs, as well as rat aortic segments, than AAV2, but the *in vivo* long-term performance of AAV vectors was not further investigated.[5] In addition, AAV7 and AAV8 were verified to transduce ECs poorly, but transgene expression could be enhanced by proteasome inhibitors.[6]

Received 9 November 2020; accepted 28 May 2021;
https://doi.org/10.1016/j.omtm.2021.05.015.

Correspondence: Lin Yang, Department of Cardiology and Laboratory of Gene Therapy for Heart Diseases, State Key Laboratory of Biotherapy, West China Hospital, Sichuan University and Collaborative Innovation Center for Biotherapy, Chengdu, Sichuan, China.
E-mail: yanglin0@hotmail.com

 © 2021 The Author(s).
This is an open access article under the CC BY license (http://creativecommons.org/licenses/by/4.0/).

The AAV capsids were composed of the VP1, VP2, and VP3 proteins at a ratio of 1:1:10, which dictated the tissue tropism of the AAV vector. Genetic engineering of the capsid gene has been verified to be very efficient in enhancing AAV transduction in various tissues, including ECs. Earlier work on rational design of AAV capsids was exemplified by insertion of specific peptides from phage display on the surface of the capsid structure, usually at site 587. The modified AAV vectors showed improved transduction of human vascular ECs independent of the natural glycan receptor of AAV2[4] or enhanced uptake of virions in the vena cava with selective transgene expression *in vivo*.[7] In addition to rational design, the potential of capsid engineering was magnified by directed evolution involving random diversification of the AAV capsid gene followed by intentional selection on a specific biological platform. For instance, DNA shuffling among AAV serotypes followed by biopanning in a mouse model was successfully performed to engineer AAV to efficiently target the myocardium with remarkably reduced liver infection.[8] Furthermore, random seven-amino-acid peptides were inserted after arginine 588 of the AAV2 capsid with the resulting libraries selected in *in vitro* cell or *in vivo* mouse models in the context of the capsid to enhance AAV transduction to human coronary artery ECs[9] or mouse brain microvasculature ECs.[10]

These successful cases motivated us to employ a directed evolution approach to tailor AAV for optimized transduction of cardiovascular ECs. AAV libraries based on DNA shuffling and random peptide display were combined for screening in a transgenic mouse model with AAV variants enriched in ECs isolated by flow cytometry and polymerase chain reaction (PCR). Characterization of these vectors revealed their remarkably improved transduction of cardiovascular ECs and reduced liver infection in a mouse model. Significantly, persistent transgenic expression was detected in mouse cardiac ECs *in vivo* over 4 months. Furthermore, endothelium-specific transgene overexpression of endothelial nitric oxide synthase (eNOS) mediated by one of these vectors partially improved cardiac function in a mouse model of myocardial infarction. Nevertheless, the numbers of ECs transduced by engineered AAVs *in vivo* were still relatively limited, which might be related to the moderate therapeutic efficacy in the following gene therapy experiment. All this work demonstrated the potential of directed evolution for engineering AAV vectors to be used for cardiovascular gene therapy.

RESULTS

At the beginning of this work, it remained a challenge to isolate AAV variants from a specific cell type of an intact organ. Fortunately, AAV successfully evolved in a transgenic mouse model in which AAV variants enriched in mouse retina photoreceptors were isolated by fluorescence-activated cell sorting (FACS) followed by PCR amplification.[11] A similar strategy was adopted to evolve AAV targeting cardiovascular ECs *in vivo* in this study.

Selection of AAV variants enriched in cardiovascular ECs *in vivo*

AAV libraries from DNA shuffling and random peptide display have both been successfully applied in the evolution of AAV with novel tissue tropism.[8,9,11,12] To increase the probability of obtaining optimized AAVs, we used a DNA shuffling library from AAV serotypes 1, 2, 3B, 4, 6, 7, 8, and 9 and a peptide display library based on the AAV2 capsid gene in the same ratio in this study. The Tie2-green fluorescent protein (GFP) transgenic mouse was selected as the model for library screening since its ECs with GFP transgene expression were ready for FACS isolation.[13] 2 weeks after library infusion, mouse hearts were collected for ECs isolation and genomic DNA extraction. The AAV capsid genes were recovered by PCR to construct plasmid and virus libraries for the second screening in Tie2-GFP mice (Figure 1A). After each round of screening, a number of capsid gene clones were picked randomly for Sanger sequencing. Two AAV variants named EC71 and EC73 emerged with the highest gene frequencies of 10% and 63%, respectively, in cardiac ECs after the second screening (Figure 1B). Intriguingly, EC71 showed a higher "enrichment score"[14] (0.98) than EC73 (0.92), which might reflect the more efficient accumulation of EC71 from the first to the second rounds of screening. Both of these AAV variants possessed the AAV2 capsid scaffold with a seven amino acid peptide insertion after arginine 588 with no mutations or recombination. The peptide was AEGDVAR for EC71 and NHPPGGV for EC73, with no consensus shown between these two variants. Based on these data, the AAV variants EC71 and EC73 were selected for further characterization of their tissue tropism.

EC71 and EC73 showed enhanced transduction and improved specificity in cardiac ECs in mice after systemic administration

EC71 and EC73 capsid genes were used for packaging recombinant vectors harnessing luciferase gene for comparison of their tissue tropism side by side with AAV2 and AAV1. AAV1 was selected as the control because it was reported to be superior to other AAV serotypes in the transduction of ECs both *in vitro* and *in vivo*.[5] The endothelial-specific fms-like receptor tyrosine kinase (Flt1) promoter was selected for regulation of transgene expression so that the luciferase activity in ECs could be detected conveniently by enzyme assay.[15] Furthermore, this promoter did not show strong preferentiality among vasculatures from different mouse organs,[16] which facilitated the examination of the tissue specificity of different AAV capsids. At 2 weeks post-intravenous-injection in young adult C57B6 mice, luciferase activities and vector DNA copy numbers in various tissues were analyzed. Consistent with a previous report, AAV1 mediated approximately 2-fold more effective endothelial transduction than AAV2 in both the heart and liver, with transgene expression mainly detected in the latter organ (Figure 2A). In contrast, EC71 and EC73 vectors showed transgene expression in ECs from the heart, liver, brain, and lung. Furthermore, these vectors showed markedly enhanced endothelial transduction in the heart and significantly reduced endothelial transduction in the liver in comparison to the natural AAV serotypes. With AAV1 as a control, the transduction of cardiac ECs was increased 8.8-fold for EC71 and 6.3-fold for EC73. In contrast, liver ECs infection was reduced 8.3-fold for EC71 and 8.9-fold for EC73. Data from the vector genomic distribution of these four AAVs among mouse tissues were fundamentally consistent with these results (Figure 2B).

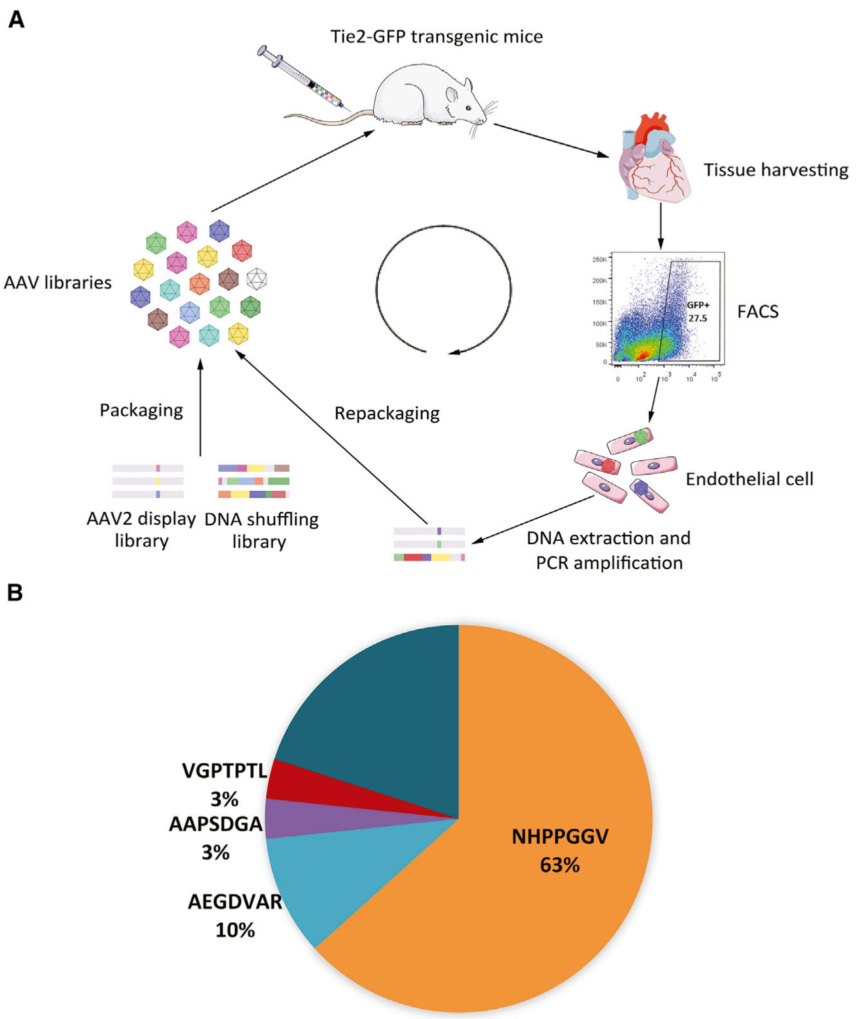

Figure 1. Directed evolution to target AAV to cardiovascular endothelial cells (ECs)

(A) Schematic of the construction and screening of AAV libraries. Two libraries were created: an AAV shuffled library and a random 7-mer insertion library. They were mixed in equal parts and injected intravenously into adult transgenic Tie2-GFP mice. 2 weeks after injection, hearts were harvested, and ECs were dissociated using collagenase II treatment, followed by FACS isolation of endothelial cells. Viral *cap* genes from the isolated cells (representing the AAV variants from the library that successfully transduced ECs) were then PCR-amplified from genomic extractions for cloning and repackaging. (B) Sequence analysis of endothelium-enriched AAV variants. Sequencing of evolved variants revealed convergence in the selected viral pools. All sampled variants but some identical to the natural serotypes originated from the AAV2 seven-amino-acid library. 10% of these clones contained the 7-mer motif (AEGDVAR), and 63% of them contained the motif (NHPPGGV). They were designated EC71 and EC73, respectively, for further characterization.

AAV1 and AAV2 showed obvious focused transduction in mouse liver by vector copy numbers. However, EC71 and EC73 were significantly detargeted from the liver, with 23.3-fold and 28.5-fold reduction in genomic copy numbers in comparison to AAV1, respectively. In contrast, the vector genomic distribution of EC71 and EC73 in the mouse heart was very similar to the vector genomic distribution of EC71 and EC73 in AAV1, with slight increases of 1.5-fold and 1.4-fold, respectively. These results inferred an interesting mechanism for endothelial transduction by EC71 and EC73, i.e., a strong tendency to detarget from the liver by reduced receptor binding and enhanced transduction of cardiac ECs, plausibly from more efficient intracellular trafficking or capsid uncoating.

It was unexpected that EC71 and EC73 were significantly detargeted from the liver. Considering its relevance to vector safety, the tissue specificity of the four vectors was further assessed by calculation of the ratios of heart versus liver transgene expression. While the heart versus liver ratios of AAV1 and AAV2 were 1:31 and 1:30, respectively, this ratio was improved to approximately 2:1 for both EC71 and EC73 (Figure 2C). Consistently, the ratios of heart versus liver vector DNA copy numbers also showed a trend similar to the luciferase activities among the four AAVs (Figure 2D). These data thus demonstrated improved tropism of EC71 and EC73 to the cardiac endothelium and much reduced infectivity of the liver endothelium.

These four AAV vectors were further used to deliver the GFP reporter gene to visualize their tissue tropism. The ubiquitous cytomegalovirus (CMV) promoter was used in this study to increase the strength of transgene expression. However, AAV1 preferentially transduced cardiomyocytes in the heart, and both AAV1 and AAV2 significantly transduced hepatocytes in the liver (data not shown). Thus, identification of endothelial transduction was difficult even when the cell marker CD31 was used. Interestingly, we did still observe sporadic transgene expression from the EC71 vector in a cardiac blood vessel, which was confirmed with CD31 antibody staining (Figure S1). To further quantitate endothelial transduction, we collected mouse hearts and livers for FACS analysis of enzymatically isolated GFP-positive cells (Figure 3). Approximately 2.0% of cardiac ECs were transduced by AAV1. In comparison, EC71 and EC73 infected 2.2% and 1.2% of cardiac ECs, respectively (Figure 3C). Approximately 3.2% of liver ECs were transduced by AAV1. In contrast, this ratio was reduced to 0.7% and 1.0% for EC71 and EC73, respectively (Figure 3D). Consequently, by direct counting of transduced ECs, EC71 showed slightly increased transduction efficiency in comparison to naturally optimal AAV1. Furthermore, EC71 also demonstrated significant detargeting from liver ECs. These results further embodied the transduction

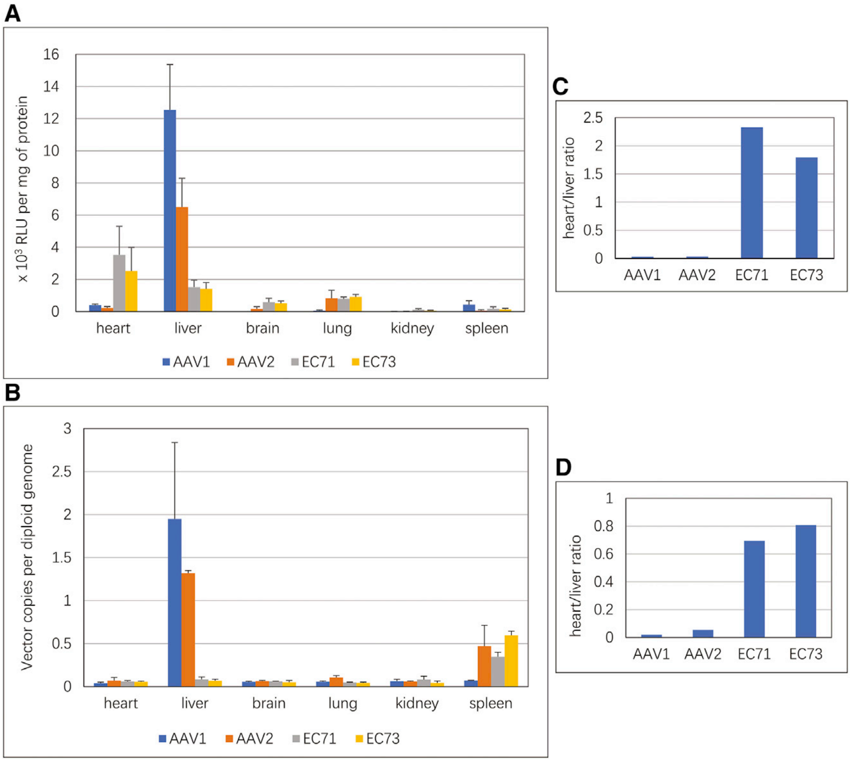

Figure 2. Luciferase activities and vector genome copy numbers in various mouse tissues after systemic administration of AAV vectors

Comparison of AAV1-, AAV2-, EC71-, and EC73-Flt1-Luc vectors in luciferase activities (A) and vector genome copy numbers (B) at 2 weeks after intravenous injection of 3×10^{11} vector genomes in adult C57B6 mice. The endothelial-specific Flt1 promoter was used in the experiment for direct measurement and comparison of transgene expression from ECs among mouse tissues and AAV vectors. Data are mean values ± SD. Heart versus liver ratio in transduction efficiency by four AAV vectors on luciferase activities (C) and vector genome copy numbers (D).

mechanism and verified EC71 as a more efficient vector for *in vivo* endothelial transduction.

Transduction of primary human and rodent ECs by EC71 vector

Since EC71 and EC73 showed enhanced transduction of cardiovascular ECs after systemic delivery, we wished to examine their direct infectivity on primary ECs *in vitro*. AAV-LacZ vectors packaged with AAV1, AAV2, EC71, and EC73 were used to infect HUVECs, and transgene expression was monitored by X-gal staining and β-gal assay. 3 days later, <1% of EC71- and EC73-infected ECs expressed the LacZ gene, but approximately 2% and 9% of the AAV1 and AAV2 cells, respectively, expressed the LacZ gene at the highest multiplicity of infection (MOI; Figure 4A). Quantitative analysis showed that the β-gal enzyme activities of AAV1-, AAV2-, and EC73-infected ECs were 68-fold, 297-fold, and 2-fold, respectively, of that of EC71 (Figure 4B).

Since HUVECs represent an interesting *in vitro* endothelial model of human origin, whether the distinct performance of four AAV vectors on these cells or on *in vivo* mouse cardiac ECs originates from interspecific differences remains a question. We thus referred to two rodent *in vitro* cardiac ECs models, primary rat myocardium microvascular ECs (RMMVECs) and mouse myocardium microvascular ECs (MMMVECs). 5 days after infection, <1% of EC71- and EC73-infected RMMVECs expressed the LacZ gene; in contrast, approximately 3% and 5% of the AAV1 and AAV2 cells, respectively, expressed the LacZ gene at the highest MOI (Figure 4C). Quantitative analysis showed that the β-gal enzyme activities of AAV1-, AAV2-, and EC73-infected RMMVECs were 185-fold, 250-fold, and 1-fold that of EC71-infected RMMVECs (Figure 4D). Similarly, <1% of EC71- and EC73-infected MMMVECs expressed the LacZ gene; nonetheless, approximately 2% and 6% of the AAV1 and AAV2 cells, respectively, expressed the LacZ gene at the highest MOI (Figure 4E). Quantitative analysis showed that the β-gal enzyme activity of AAV1-, AAV2-, and EC73-infected MMMVECs was 51-fold, 135-fold, and 1-fold, respectively, of the β-gal enzyme activity of EC71 (Figure 4F). These data clearly indicated that EC71 and EC73 infectivity in ECs was significantly lower than the EC71 and EC73 infectivity in ECs of AAV1 and AAV2 *in vitro*. Furthermore, the different performances of these vectors originated from the distinct biological properties of ECs *in vitro* and *in vivo* but not their interspecies differences.

EC71 and EC73 mediated more effective and stable transgene expression in ECs *in vivo*

It remained an attractive aim to realize long-term transgene expression in cardiac ECs *in vivo*; thus, we sought to investigate the persistence of endothelial transduction by EC71 and EC73 after systemic administration in mice. AAV1 was used as the control because its endothelial transduction was higher than AAV2. Transgene expression in ECs was monitored in the long term for up to 4 months. Transgene expression in mouse heart mediated by the three vectors all kept rising during the whole time range, with the transgene expression of EC71 and EC73 4.1-fold and 4.0-fold higher than AAV1 at 4 months after vector administration (Figure 5A). In the liver, however, the transgene expression from three vectors demonstrated a different tendency, i.e., continuing to rise and reaching a peak at 1 month following gene delivery but gradually decreasing by 2 months after vector administration (Figure 5B). Interestingly, the two modified vectors continued to display luciferase activity that was significantly lower than the luciferase activity of AAV1, with the transgene expression of EC71 and EC73 at the peak level (1 month) 19-fold and 19.2-fold lower than the transgene expression of AAV1, respectively. The transgene expression of all three vectors

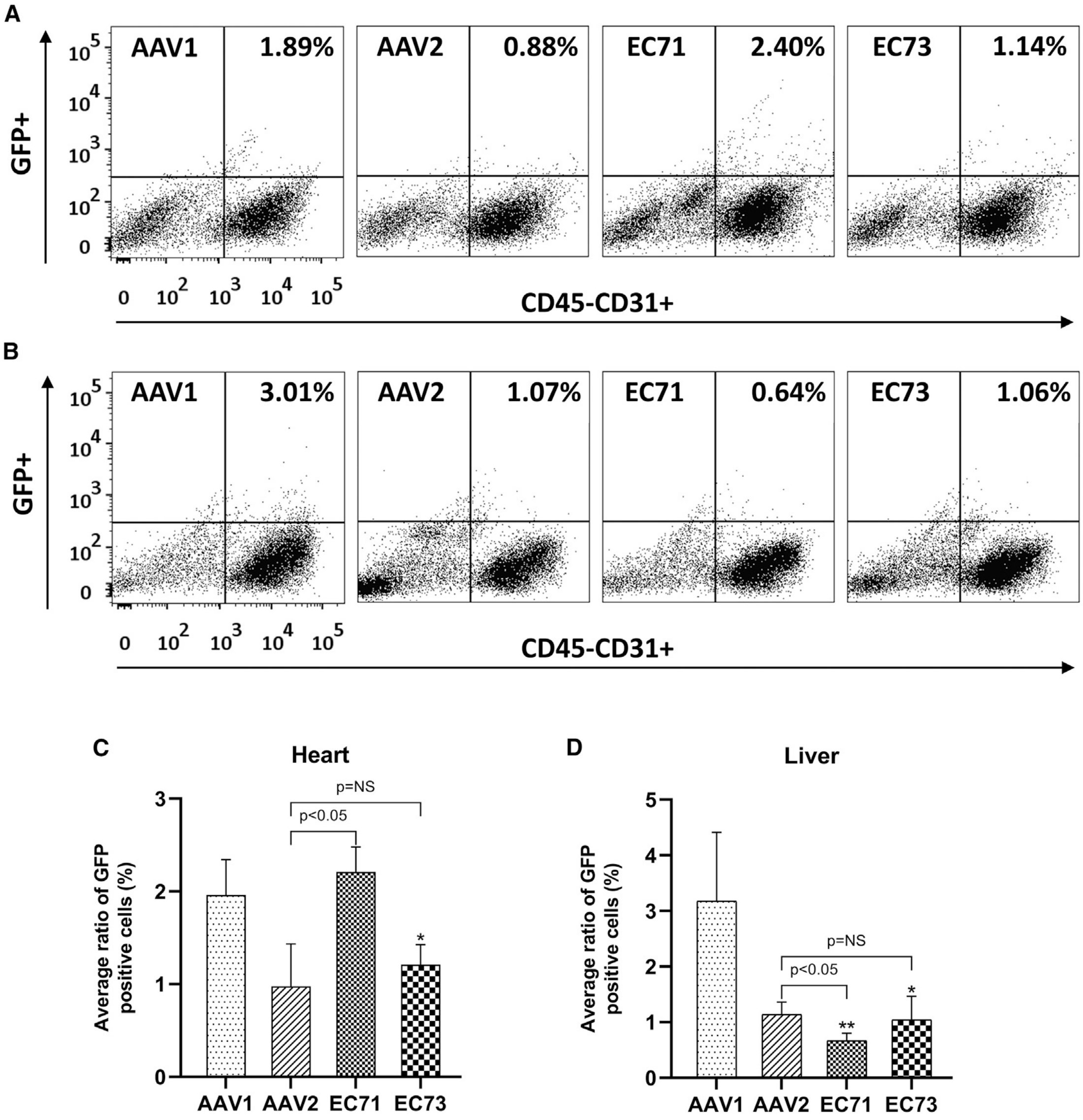

Figure 3. Tracking of AAV-transduced ECs in mouse organs

AAV1-, AAV2-, EC71-, and EC73-CMV-GFP vectors were injected into young adult C57B6 mice at a dose of 3×10^{11} vector genomes. After 2 weeks, GFP transgene expression within the ECs was tracked among mouse organs by flow cytometry. (A and B) Representative FACS graphs of isolated GFP^+ cells (upward scatter) against the ECs marker CD45-$CD31^+$ (rightward scatter) within mouse hearts (A) and livers (B). (C and D) The percentage of GFP-positive ECs was further plotted from the four mouse groups for side-by-side comparison. Data are mean values ± SD. * and **, significantly different from AAV1 by Student's t test ($p < 0.05$ and $p < 0.01$, respectively). NS, nonsignificant.

remained stable in brain ECs over the whole time range but was relatively low (Figure 5C). With respect to the lung, all three vectors showed a gradual increase in transgene expression in the first 2 months but decreased remarkably, especially AAV1 thereafter (Figure 5D). Significantly, the lung endothelial transduction of EC71 and EC73 was 2.3-fold and 1.8-fold higher than the lung endothelial transduction of AAV1, respectively, at the peak level (2 months). These results showed persistent transgene expression mainly in cardiac ECs by the EC71 and EC73 vectors, which motivated us to further investigate their application to cardiovascular gene therapy.

Gene therapy application of EC71 in a chronic heart failure mouse model

Because of the pivotal role of nitric oxide in vascular biology, eNOS has been an attractive target for endothelium-targeted gene therapy.[17] Furthermore, the positive effects of eNOS expression on ventricular dysfunction and remodeling have been verified in transgenic mouse models after myocardial infarction.[18,19] Considering its higher transduction efficiency in cardiac ECs *in vivo*, we next investigated the utility of EC71 for the delivery of eNOS for gene therapy of chronic heart failure in a mouse model of myocardial infarction.

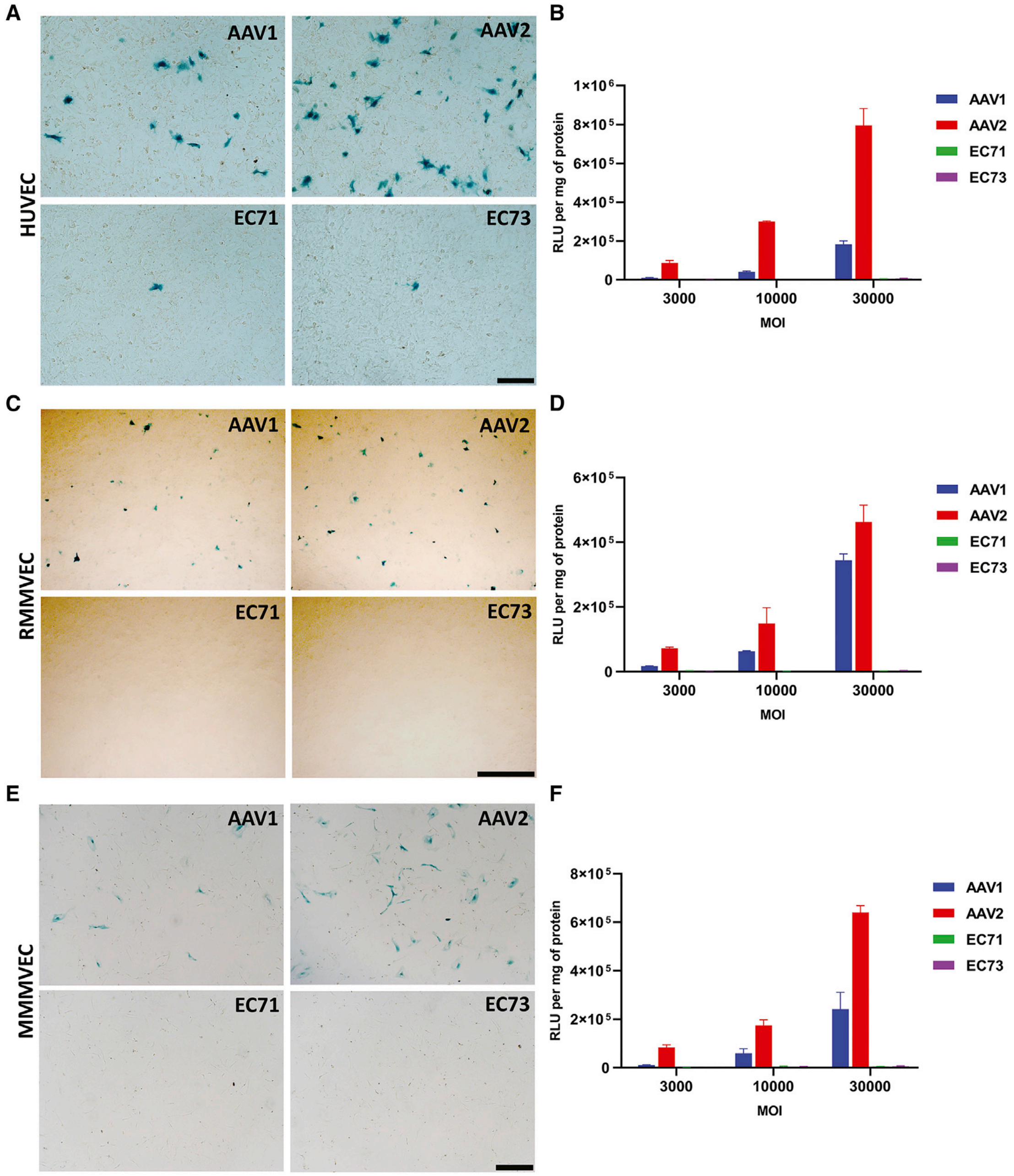

Figure 4. Comparison of gene transfer efficiency in HUVECs, RMMVECs, and MMMVECs

Representative X-gal staining in HUVECs (A), RMMVECs (C), or MMMVECs (E) after transduction by AAV1-, AAV2-, EC71-, or EC73-CMV-LacZ vectors. (A) AAV vectors were inoculated onto HUVECs at a multiplicity of infection (MOI) of 30,000. Cells were fixed for X-gal staining 72 h later. Scale bar, 100 μm. (C) AAV vectors were inoculated onto RMMVECs at an MOI of 30,000. Cells were fixed for X-gal staining 5 days later. Scale bar, 250 μm. (E) AAV vectors were inoculated onto MMMVECs at an MOI of 30,000. Cells were fixed for X-gal staining 5 days later. Scale bar, 100 μm. Quantitative β-gal activities in HUVECs (B), RMMVECs (D), and MMMVECs (F) after infection with four AAV vectors at different MOIs. Data are mean values ± SD.

In retrospect of the previous references, the promoter regulating eNOS expression was found to be possibly crucial for gene therapy, with the native eNOS promoter showing better therapeutic efficacy.[20,21] In this regard, two promoters, Flt-1 and native eNOS, were compared in terms of their strength and tissue specificity in the regulation of transgene expression in the endothelium from different organs (Figure S2). As a result, the Flt-1 promoter showed stronger transgene expression in cardiac ECs than the eNOS

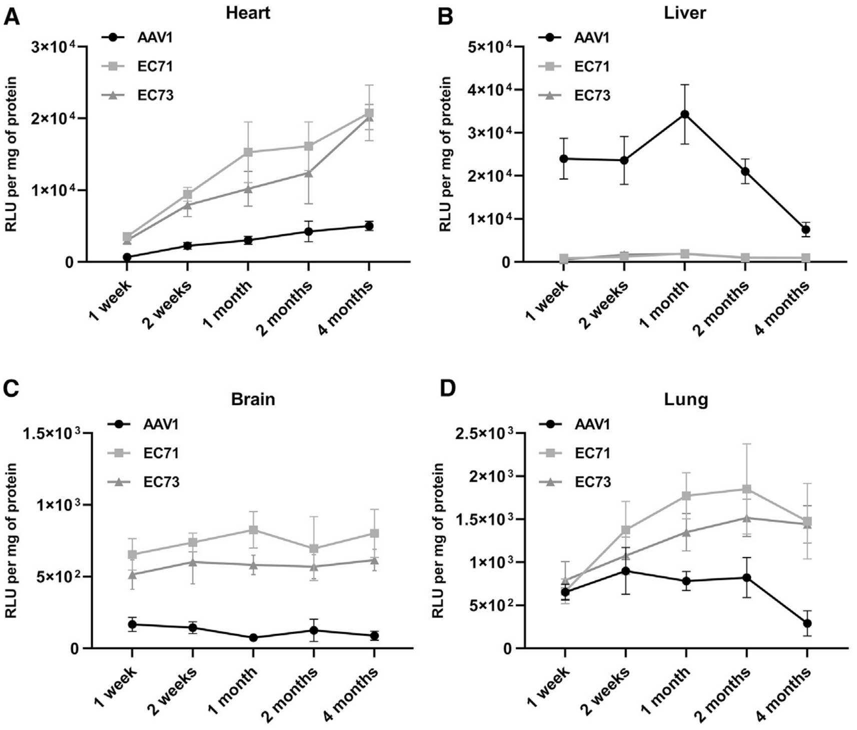

Figure 5. Kinetics of luciferase activity in C57B6 mice
Male adult C57B6 mice were injected intravenously with 3×10^{11} vector genomes of AAV1-, EC71-, and EC73-Flt1-Luc, and luciferase activity was measured by enzyme assay at different time points in mouse heart (A), liver (B), brain (C), and lung (D). Four mice were injected as a group corresponding to an AAV and a specific time point. The usage of the Flt-1 promoter ensured the detection of endothelial-specific transgene expression. Each data point corresponded to the mean of four animals. Circles, AAV1; squares, EC71; triangles, EC73. Data are mean values ± SD.

promoter, especially at the lower vector dose. However, the eNOS promoter displayed higher tissue specificity to heart blood vessels. Based on these data, both of these promoters were employed for delivery of eNOS in further gene therapy studies.

A total of 1×10^{12} vector genomes of EC71-Flt1-eNOS, EC71-eNOS-eNOS, EC71-Flt1-GFP, or PBS were injected into 7-week-old male C57B6 mice via the tail vein (Figure 6A). 2 weeks later, coronary arteries of the groups with vector treatment were permanently occluded by left anterior descending (LAD) artery ligation. After another 60 days, cardiac function of the mice was assessed by echocardiography and ventricular pressure catheters. eNOS expression and activity in mouse organs were further detected by western blot and enzyme assays.

Interestingly, no apparent alteration in eNOS expression was observed among the four mouse groups in the four internal organs, including the heart, liver, brain, and lung, by western blot analysis (Figure 6B). However, eNOS activity was significantly increased in the cardiac ECs of the Flt1-eNOS and eNOS-eNOS groups in comparison to the cardiac ECs of the Flt1-GFP group (Figure 6C). Similar improvement in eNOS activity was also observed in the pulmonary ECs of the Flt1-eNOS group compared with the Flt1-GFP group (Figure 6C). These data inferred the higher sensitivity of the enzyme assay in eNOS detection in comparison to immunoblotting. The moderate gene transfer efficiency of the EC71 vector in combination with a stronger endothelial-specific promoter resulted in a detectable increase in eNOS expression in cardiac and pulmonary endothelium.

60 days after surgery, coronary artery occlusion induced profound effects on ventricular dimensions (Figures 6D and 6E). Namely, left ventricular chamber diameters were significantly ($p < 0.01$) greater in diastole (LVIDd) and systole (LVIDs) in the insulted hearts than in the sham hearts. The differences between GFP- and eNOS-treated mice were not statistically significant (p = NS). Fractional shortening was also significantly ($p < 0.01$) lower in the three heart failure groups than in the sham group (Figure 6F). Importantly, Flt1-eNOS-treated mouse hearts exhibited an evidently but nonsignificantly greater extent of fractional shortening than the GFP group (20.5% ± 6.4% versus 15.0% ± 3.7%). The left ventricular chamber thickness in diastole (LVPWd) did not significantly change among the groups (Figure 6G). Similarly, the infarcted groups also exhibited significantly ($p < 0.05$) diminished left ventricular systolic pressure (LVSP) and end diastolic pressure (LVEDP) compared with the sham group (Figures 6H and 6I). Interestingly, evident but not significant improvement in LVEDP was observed for the Flt1-eNOS and eNOS-eNOS groups compared with the sham group. Furthermore, both maximum (+) and minimum (–) dP/dt were significantly attenuated in infarcted hearts compared with sham hearts (Figures 6J and 6K). However, evident but not significant improvement was observed for the Flt1-eNOS group in minimum dP/dt compared with the GFP group (−3,068 ± −1,065 mm Hg/sec versus −2,122 ± −211 mm Hg/sec). All these functional data implied that eNOS overexpression from the combination of the EC71 vector and Flt-1 promoter contributed to the partial recovery of cardiac systolic and diastolic functions in mice with heart failure.

Influence of peptide insertion on receptor usage of AAV vectors

AAV2 adopts heparin sulfate proteoglycan and AAVR as its glycan and protein receptors, respectively, for cellular transduction.[22,23] We were curious whether EC71 and EC73 still maintained their receptor usage after peptide insertion. As shown in Figure 7A, AAV2 demonstrated a strong tendency to bind heparin, with most of the viruses eluted when a high concentration of NaCl was applied. As direct derivatives of AAV2, however, EC71 and EC73 showed severe attenuation of heparin binding ability distinct from the heparin binding

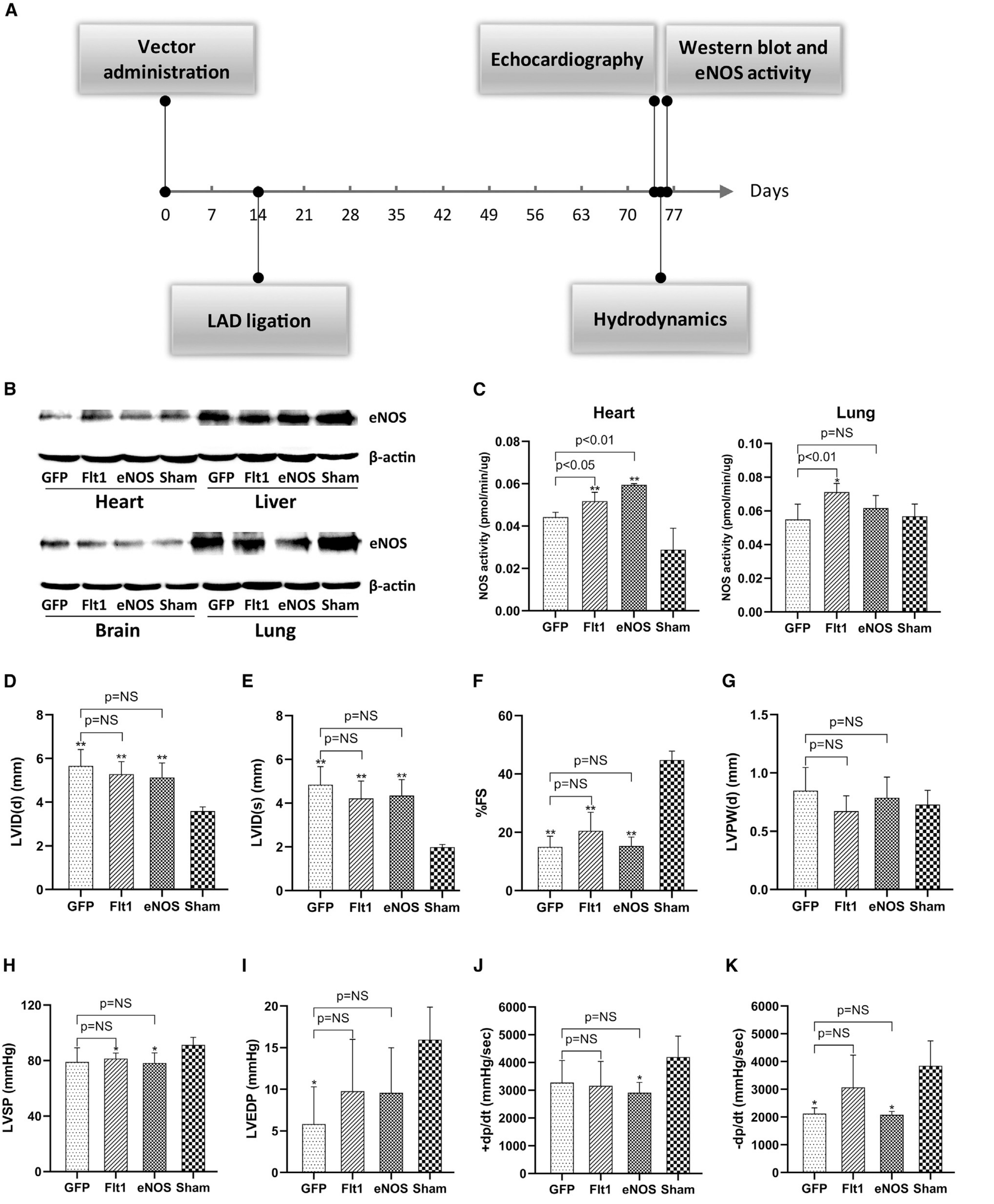

(legend on next page)

ability of the parental AAV2 but very similar to the heparin binding ability of AAV1. Furthermore, both AAV1 and AAV2 depended on AAVR for transduction of HeLa cells, which was inferred by the markedly reduced infectivity of these cells after AAVR knockout (KO) by use of CRISPR-Cas9 gene editing (Figures 7B and 7C). In fact, EC71 and EC73 showed relatively reduced transduction of wild-type HeLa cells in comparison to AAV1 and AAV2. Intriguingly, these two vectors were further heavily attenuated and barely detected in AAVR-KO HeLa cells (Figures 7B and 7C), while AAVR expression was completely depleted in these cells, as indicated by western blot analysis (Figure S3). Taken together, these results inferred that the endothelial-targeting EC71 and EC73 vectors most likely retained the employment of AAVR as their proteinaceous receptor but were devoid of heparin sulfate binding during their cellular attachment.

DISCUSSION

Because of its central role in vascular pathogenesis, the endothelium is an attractive therapeutic target for cardiovascular disease.[1] However, very few studies have considered endothelial-specific transgene expression as a therapeutic modality for cardiovascular disease. With the eNOS gene as an example, its beneficial effects have been universally verified for various cardiovascular diseases, including hypertension,[2,3,24] atherosclerosis,[20,21] and myocardial infarction.[18,19] However, the therapeutic effects were transient when plasmid or adenovirus vectors were used,[2,3,24] or the therapeutic potential was investigated only in transgenic animals where persistent transgene expression in the endothelium could be realized.[18–21] All these studies urged the development of novel gene therapy vehicles for efficient and stable delivery of therapeutic genes into the cardiac endothelium *in vivo*.

In this context, we sought to genetically engineer an AAV vector to improve its transduction in cardiac ECs *in vivo*. Naturally, AAVs can infect ECs with relatively low efficiency.[4,5] After modification, the EC71 and EC73 vectors showed 8.8-fold and 6.3-fold increases in the transduction of cardiac ECs in mice in comparison to naturally optimized AAV1 (Figure 2A). Furthermore, these two vectors displayed significantly reduced liver infection, and their heart versus liver ratio in transgene expression increased 73-fold and 56-fold, respectively, in comparison to AAV1 (Figure 2C). In addition, when used for long-term gene transfer, EC71 and EC73 showed persistently higher transgene expression in mouse cardiac ECs (approximately 4-fold higher than that of AAV1) and markedly lower transgene expression in the liver endothelium (approximately one-twentieth of the transgene expression in the liver endothelium of AAV1; Figures 5A and 5B). All this work demonstrated the improved efficiency and safety of EC71 and EC73 for use in cardiac endothelium-targeted gene transfer.

However, when used for delivery of eNOS into cardiac ECs of mice with heart failure, the EC71 vector showed moderately improved transgene expression and activity (Figures 6B and 6C). Furthermore, the improvement of cardiac systolic and diastolic function was observable but not significant in comparison to the GFP control (Figures 6D–6K). Persistent transgene expression in the cardiac endothelium was confirmed by the use of a reporter gene in normal mice for at least 4 months (Figure 5A); thus, the turnover of the endothelium might not be the cause of marginal improvement in eNOS expression.[25] As shown by flow cytometry analysis, the number of positively transduced cardiac ECs was not significantly increased by EC71 in comparison to AAV1 (Figure 3), which partially indicated the insignificantly improved eNOS transgene expression. Consequently, the increase in vector dose and adoption of a relatively local delivery route might be the solution to the moderate gene transfer efficiency of our vectors.[26] Furthermore, cardiac function was partially improved in both systolic (fraction shortening, Figure 6F) and diastolic stages (LVEDP and –dP/dt, Figures 6I and 6K). Considering the primary effects of nitric oxide on vasodilation, the improvement of cardiac systolic function might come after effective ventricular diastole. Last, the maintenance of normal pulmonary circulation inferred by the higher lung endothelial eNOS activity might also partially account for the improved cardiac function in Flt1-eNOS mice (Figure 6C).

Regarding the directed evolution method, some seminal work has been done to target AAV to the endothelium both *in vitro*[9] and *in vivo*.[10] The advantage of this work was the adoption of a transgenic mouse model for *in vivo* screening with AAV variants enriched in cardiac ECs ready for isolation by FACS. As a result, the two selected vectors showed significantly improved transduction efficiency and specificity in cardiac ECs *in vivo* (Figure 2) and remarkably decreased infection of primary ECs *in vitro* (Figure 4) in comparison to natural AAV serotypes. These data further validated the vital importance of biological models in the directed evolution of AAV vectors. Unfortunately, the selected AAV vectors were not completely optimized for *in vivo* endothelium-targeted gene therapy applications in this study. The following designs might be integrated into tailoring AAV vectors in the future. First, parental capsid sequences should be carefully trimmed to balance their homology, and structural information could

Figure 6. Systemic delivery of eNOS into mouse cardiac ECs for treatment of myocardial infarction
Overall design of the gene therapy study (A). A total of 1×10^{12} vector genomes of EC71-Flt1-eNOS, EC71-eNOS-eNOS, EC71-Flt1-GFP, or PBS were injected into 7-week-old male C57B6 mice via the tail vein. Myocardial infarction was induced in the vector-treated mouse groups via permanent ligation of the left anterior descending artery 2 weeks later. The PBS-treated group underwent a sham operation. 60 days after surgery, the mice were sacrificed for detection of transgene expression by western blot (B) and assessment of eNOS activity (C) in four internal organs. eNOS activity was determined by a series of reactions and color development with Griess Reagent. Furthermore, mouse cardiac function was assessed by echocardiography (D–G) and a pressure transduction catheter (H–K). LVID(d), left ventricular internal diameter end diastole; LVID(s), left ventricular internal diameter end systole; %FS, percentage of fractional shortening; LVPW(d), left ventricular posterior wall dimension end diastole; LVSP, left ventricular systolic pressure; LVEDP, left ventricular end diastolic pressure. * and **, significant compared with sham by Student's t test ($p < 0.05$ and $p < 0.01$, respectively). NS, nonsignificant compared with EC71-Flt1-GFP. Data are mean values ± SD.

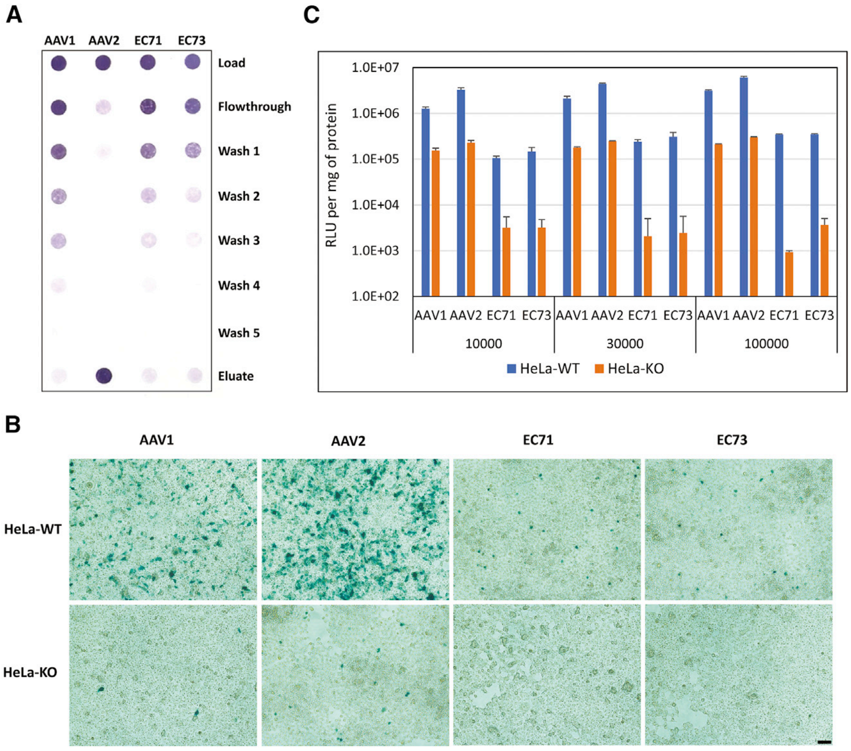

Figure 7. Receptor usage of endothelial-targeting AAV vectors

(A) Heparin binding profile of AAV1, AAV2, EC71, and EC73. Recombinant AAV vectors were loaded onto heparin-conjugated affinity columns, and the collections were titrated by dot blots. (B and C) EC71 and EC73 demonstrated AAVR-dependent cellular transduction *in vitro*. (B) AAV1-, AAV2-, EC73-, or EC73-CMV-LacZ vectors were inoculated on wild-type (HeLa-WT) or AAVR-knockout HeLa cells (HeLa-KO) at an infection multiplicity of 100,000. Cells were fixed for X-gal staining 72 h later. Scale bar, 100 μm. (C) Quantitative detection of β-gal activities of HeLa-WT and HeLa-KO cells after infection of recombinant AAV vectors at the indicated multiplicity. Data are mean values ± SD.

be integrated into homologous recombination to construct a more potent DNA shuffling library.[27,28] Furthermore, random peptides could be presented on capsids of different AAV serotypes to expand the application of AAV peptide display libraries.[29] Last, humanized animal or nonhuman primate models could be applied to obtain clinically relevant AAV vectors.[12,30] Integration of all these technical advancements might facilitate the evolution of AAV vectors for *in vivo* endothelium-targeted gene therapy in the future.

Both AAV variants selected in this study were based on the AAV2 capsid scaffold with peptide insertion after the 588 site. The lack of consensus between these peptides (AEGDVAR versus NHPPGGV) inferred their different transduction mechanisms. However, further studies from vector genomic distribution (Figures 2C and 2D) and flow cytometry analysis (Figures 3A–3D) supported a similar rationale for their enhanced endothelial transduction efficiency and specificity, i.e., significantly increased intracellular trafficking or capsid uncoating efficiency in cardiac ECs and markedly reduced liver ECs affinity. A minor contradiction to this hypothesis was that EC71 also showed improved cardiac endothelium affinity in comparison to parental AAV2, as implied by flow cytometry data (Figures 3A and 3C).

Finally, the transduction mechanism of AAV serotypes or variants remains largely unknown. Various glycans were deduced to be responsible for attachment of AAV serotypes to target cells.[31] Furthermore, AAVR was verified to be essential for cellular entry of most natural AAV serotypes, except for AAV4.[23] EC71 and EC73, in comparison to their parental serotype AAV2, lost or severely attenuated their heparin binding capacity. However, they still retained their usage of AAVR for cellular entry. Significantly, this phenomenon has been very prevalent in different AAV vectors engineered from natural serotypes, including those from SCHEMA-based homologous recombination,[28] from peptide insertion correspondent or adjacent to AAV2 588 site,[32,33] and from ancestral reconstruction.[32] These results strongly inferred a common evolutionary process for both the natural AAV serotypes and engineered AAV variants. In other words, they evolved to adopt various primary receptors for cell attachment, mainly the different glycans on the cellular surface such as heparin sulfate and sialic acids. In contrast, they tended to be conservative in the usage of proteinaceous receptors for cellular entry, which might be dictated by conservatism in the exterior structure of their capsids.[34,35]

In conclusion, we attempted to tailor AAV vectors targeting cardiac ECs *in vivo* by directed evolution in this study. The vectors selected from a transgenic mouse model showed enhanced transduction efficiency and specificity in cardiac ECs *in vivo*. Importantly, this might be the first report on viral vectors capable of mediating stable transgene expression in the cardiac endothelium in a mouse model. One of these vectors exhibited partial therapeutic efficacy in a mouse model of heart failure. The further optimization of AAV vectors for endothelium-targeted gene transfer might ultimately benefit the clinical gene therapy of cardiovascular disease.

MATERIALS AND METHODS

Generation of AAV libraries from DNA shuffling and random peptide display

Two separate AAV libraries were used for directed evolution in this study. The DNA shuffling library has been described previously for the selection of AAV vectors targeting the myocardium.[8] Homologous recombination was introduced among capsid genes of AAV serotypes 1, 2, 3B, 4, 6, 7, 8, and 9 by DNase I treatment followed by PCR reassembly.

Meanwhile, an AAV2 peptide display library was constructed following Muller et al.[9] with some modifications. In brief, the plasmid UF1-AAV8 containing the AAV2 *rep* gene and ITRs served as a backbone for cloning of the random oligonucleotides. The *Sfi*I site in the *rep* gene was deleted to generate UF1-AAV8(M) by a silent mutation introduced by PCR mutagenesis. The plasmid XX2 (a kind gift of Dr. Xiao Xiao) contained the AAV2 *cap* gene, which was modified by insertion of two incompatible *Sfi*I restriction sites after codon 588. These *Sfi*I sites, including a stuffer region, were generated by mutagenesis using the following primers: 588-For: 5′-GGCCCAGGCGGCC-ACCGCAGA TGTCAACACACAAGGC-3′; 588-Rev: 5′-TTGGCCTCTCTG-GCCT CTCTGGAGGTTGGTAGATAC-3′; Amp-1: 5′-CGTTGTCAGAAG TAAGTTGGCCGCA-3′; Amp-2: 5′-ATCGGAGGACCGAAGGAGC TAACCG-3′. The altered plasmid clone after sequence verification was designated XX2(M). The modified AAV2 *cap* gene was amplified and subcloned into the UF1-AAV8(M) plasmid by using *Hind*III/ *Not*I digestion to generate UF1-AAV2(M) as a plasmid backbone for insertion of random peptides. The synthesized oligonucleotide encoding random peptides had two different *Bgl*I restriction sites on each end: 5′-CAGTCGGCCAGAGAGGC$(NNK)_7$GCCCAGGCGGCTGA CGAG-3′. The second strand was synthesized by using the primer 5′-CTCGTCAGCCGCCTGG-3′ and Phusion high-fidelity DNA polymerase (Thermo F-530). The double-stranded insert was digested by *Bgl*I and ligated into the *Sfi*I-treated UF1-AAV2(M) backbone in a 15:1 molar ratio. Ligated plasmids were transformed into electrocompetent *DH10B* bacteria using a Multiporator (Eppendorf). Colonies (1.8×10^4 and 2.4×10^4) were collected for DNA shuffling and peptide insertion libraries, respectively. The diversity of the plasmid library was determined by Sanger sequencing of 40 bacterial clones. Approximately 60% and 100% of the clones encompassed the correct shuffled or peptide-inserted capsid genes, respectively. Library plasmids were harvested from transformed bacteria and purified using cesium chloride ultracentrifugation. The DNA shuffling and random peptide display plasmid libraries were packaged into wild-type AAV2 capsids (AAV transfer shuttles) by transfecting four 15-cell culture dishes.[9] The resulting AAV library shuttles were used to infect 293 cells in thirty 15-cm cell culture dishes at a MOI of 1,000, and cells were superinfected with Ad5 to ensure that complete cytopathic effects were observed 36 h later when AAV libraries were harvested. The AAV libraries were purified by three freeze/thaw cycles followed by two rounds of cesium chloride density gradient centrifugation.[36] The virus titers at the genomic level were determined by dot blot.

In vivo screening of AAV libraries in transgenic mice

All animal protocols were approved by the West China Hospital Animal Care and Use Committee. For *in vivo* selection, 1×10^{12} genomic particles of two AAV libraries mixed in the same ratio were injected into Tie2-GFP transgenic mice[13] via the tail vein. 2 weeks later, the mice were sacrificed, and the complete hearts were removed for dissociation with collagenase type 2 (Worthington) treatment followed by FACS (FACSAria III Cell Sorter, BD Biosciences) isolation of ECs. DNA was extracted, and endothelium-targeted AAV particles were amplified by PCR using the primers CAP-5′: 5′-CCCAAGCTTC-GATCAACTACGCAGACAGGTACCAA-3′ and CAP-3′: 5′-ATAAGAATGCGGCCGCAGAGACCAAAGTTCAAC TGAAACGA-3′. The PCR products were digested with *Hind*III and *Not*I and recloned into the UF1-AAV8 plasmid for production of a secondary AAV library as described earlier. This AAV library was again injected intravenously into mice for secondary screening and retrieval from cardiac ECs. After each screening, tens of random colonies were sequenced to evaluate the process of screening and identify the endothelium-enriched AAV variants.

Tissue tropism of AAV vectors in mice after systemic administration

The AAV-Flt1-luc plasmid was constructed by replacing the CMV promoter in the AAV-CMV-luc plasmid with the Flt-1 promoter[15] (−748/+284) amplified from the Flt1-GFP plasmid (a kind gift from Dr. Luis Vaca). The EC71 and EC73 capsid genes were used to package Flt1-luciferase and CMV-LacZ reporter vectors for comparison with AAV1 and AAV2 packaged vectors. The viruses were produced by triple-plasmid cotransfection of 293 cells by the calcium phosphate method and purified by two rounds of cesium chloride density-gradient centrifugation.[36] A total of 3×10^{11} vector genomes of reporter vectors containing the Flt1-luciferase gene were injected intravenously into 7-week-old male C57BL/6J mice for systemic gene delivery and expression. The heart, liver, brain, lung, kidney, and spleen of the mice were collected 2 weeks later. Approximately 100 mg of the tissues was homogenized using a mechanical tearor (BioSpec), and reporter gene expression was monitored by luciferase assay (Luciferase Assay System, Promega). Total DNA was extracted from mouse tissues by using a DNeasy Tissue Kit (QIAGEN) for quantitative detection of the vector genome copies by a TaqMan probe (Applied Biosystems) using primers (luc-F: 5′-TTGA CCGCCTGAAGTCTCTGA-3′; luc-R: 5′-ACACCTGCGTCGAAG ATGTTG-3′). The mouse glucagon gene was quantitated by primers (glu-F: 5′-AAGGGACCTTTACCAGTGATGTG-3; glu-R: 5′-ACT TACTCTCGCCTTCCTCGG-3′) as the diploid cell number reference.

Flow cytometry analysis of transgene expression

The EC71 and EC73 capsid genes were used to package CMV-GFP reporter vectors for comparison with AAV1 and AAV2 packaged vectors. A total of 3×10^{11} vector genomes of reporter vectors were injected into 7-week-old male C57BL/6J mice via the tail vein. After 2 weeks, the heart and liver were collected and digested in collagenase type I (GIBCO) at 37°C for 40 min, pipetted several times, and filtered through a 70-micron filter. After treatment with red blood cell lysis buffer (155 mM NH_4Cl, 10 mM $KHCO_3$, 0.1 mM EDTA), cells were protected from light and incubated for 30 min on ice with 1% donkey serum in PBS containing both PE-conjugated CD31 antibody (BD PharMingen, 553373) and PerCP-Cy conjugated CD45 antibody (BD PharMingen, 550994) at a 1:300 dilution. Cells were then washed three times with PBS and fixed in 2% paraformaldehyde at 4°C. Finally, the cells were washed once with PBS and analyzed for the co-localization of GFP^+ cells and ECs ($CD31^+$, $CD45^-$) by an LSRFortessa flow cytometer (BD Biosciences). Analysis was performed using FlowJo vX.

Transduction of HUVECs, RMMVECs, and MMMVECs by AAV vectors

HUVECs were prepared using the ordinary procedure.[37] RMMVECs were isolated and cultured as previously reported.[38] In brief, a 5-week-old female Sprague-Dawley rat was anesthetized intraperitoneally with pentobarbital, and its ventricle was cut into pieces of approximately 2 mm^3 for culture in a 25-cm^2 flask in DMEM supplemented with 20% fetal bovine serum. After 60 h, the tissues were discarded, and the ECs that migrated into the flask continued to grow into confluency with medium partially changed. MMMVECs were isolated by a magnetic bead sorting procedure.[39] Briefly, four 7-week-old male C57BL/6J mice were sacrificed, and their hearts were treated with collagenase type 2 (Worthington, LS004176). The cell suspension was filtered through a 40-micron filter followed by red blood cell lysis. 4 μL of rat anti-mouse CD31 antibody (BD PharMingen, 557355) and 10 μL of Dynabeads sheep anti-rat immunoglobulin G (IgG; Invitrogen, 11035) were incubated overnight at 4°C. The antibody-conjugated magnetic beads were washed three times to remove excess antibody and incubated with the dissociated cells at 4°C for 45 min. Cells attached to beads were washed five times and then resuspended in EndoGRO Basal Medium (Millipore, SCME-BM) supplemented with 10% fetal bovine serum for further culture.

24 h after preplating, EC71-, EC73-, AAV1-, or AAV2-CMV-lacZ vectors were inoculated onto HUVECs at infection multiplicities of 3,000, 10,000, and 30,000. After another 72 h, cells were fixed for X-gal staining or β-gal assay (Galacto-Light Plus β-Galactosidase Reporter Gene Assay System, Applied Biosystems) to detect transgene expression. With respect to RMMVECs and MMMVECs, the same infection multiplicities of four AAV vectors were inoculated into the cells 48 h after preplating into collagen I (BD 354236)-coated plates. After another 5 days, cells were fixed for X-gal staining or β-gal assay.

eNOS gene therapy in a mouse myocardial infarction model

The human eNOS coding sequence (GenBank Number BC069465) was codon-optimized by GeneArt before cloning into a recombinant AAV plasmid. Its expression was under regulation of the Flt-1 promoter from the AAV-Flt1-luc plasmid or the eNOS promoter (−1,033/+22)[40] from the pGL2 enhancer-F1 LUC plasmid (Addgene). To examine their therapeutic efficacy, we injected 1×10^{12} vector genomes of EC71-Flt1-eNOS, EC71-eNOS-eNOS, and EC71-Flt1-GFP into 7-week-old male C57BL/6J mice via the tail vein. After 2 weeks, permanent ligation of the LAD coronary artery was performed similar to methods described.[41] Briefly, after being anesthetized with 7.47 mg/mL ketamine and 0.51 mg/mL xylazine via intraperitoneal injection, mice were intubated with an 18-gauge catheter and ventilated with a mouse Minivent ventilator (Harvard Apparatus). Left thoracotomy was then performed through the fourth intercostal space. After the pericardium was opened, a 7-0 silk suture was passed under the LAD, and then the chest was closed in layers. An additional mouse group was injected with PBS and subjected to sham surgery. 2 months after surgery, the cardiac function of the mice was evaluated by echocardiography and hemodynamics. Then, their hearts, livers, brains, and lungs were collected and frozen before detection of eNOS expression and activity.

Western blotting and eNOS activity assay

Frozen heart, liver, brain, and lung tissues were homogenized in radioimmunoprecipitation assay (RIPA) lysis buffer (P0013B, Beyotime). 90 μg of protein (BCA Protein Assay Kit, Beyotime) was applied to each lane for 8% SDS-PAGE. Proteins were transferred to polyvinylidene fluoride (PVDF) membranes. The western blots were probed overnight at 4°C with primary antibodies mouse anti-human eNOS 1:150 (Santa Cruz, sc-376751) or mouse anti-mouse actin 1:4,000 (GenScript, A00702). Subsequently, HRP-conjugated anti-mouse IgG secondary antibody 1:2,000 (Cell Signaling, 7076) was added for 1 h at room temperature. For detection, we applied enhanced chemiluminescence (Thermo, 34577) and a MiniChemi imaging and analysis system (SageCreation).

The frozen heart and lung tissues were homogenized in cold NOS assay buffer (containing protease inhibitor cocktail). eNOS activity assays were performed by colorimetric reactions with Griess Reagent 1 and 2 with a NOS activity assay kit (Abcam, ab211083) according to the manufacturer's instructions. All measurements of eNOS activity were normalized to the total protein content measured by using the BCA protein kit (Beyotime, P0010).

Left ventricular function by echocardiography and hemodynamics

Cardiac functions were analyzed by transthoracic echocardiography with a 13-MHz probe and a VIVID i ultrasound machine (GE Health). The mice were anesthetized with 3% isoflurane. Two-dimensional targeted M-mode imaging was obtained from the short axis immediately below the level of the mitral valve. M-mode tracings were recorded, and measurements were made with the operator blinded to the mouse groups.

Mice were anesthetized with pentobarbital by intraperitoneal injection of 70 mg/kg and catheterized with a 1.4-French catheter (SPR-839, Millar Instruments). Hemodynamics were recorded by PowerLab 8/35 with Chart Pro (AD Instruments) similar to methods described.[42] Data for each animal were calculated from at least 30 cardiac cycles of chart recording.

Heparin affinity assay

A modified protocol was employed to analyze the heparin affinity of AAV variants.[43] Levels of 5×10^{10} v.g. of recombinant AAV1, AAV2, EC71, and EC73 packaging a CMV-LacZ transgene were loaded to heparin-conjugated agarose type I (Sigma) in affinity columns (Bio-Rad microspin column) in 500 μL Ringer's saline solution (RSS). Fractions were sequentially collected from flowthrough, washed with RSS five times, and eluted with RSS containing 800 mM NaCl. The number of mutant or parental AAV particles present in each fraction was determined by dot blot hybridization.

Generation and transduction of AAVR KO HeLa cells

CRISPR-Cas9 gene editing was utilized to generate AAVR KO HeLa cells as described with some modifications.[23] Oligonucleotides corresponding to the guide RNA (gRNA) sequences were synthesized and directly cloned into the Cas9-expressing pX330 plasmid (generated by the Zhang lab): sgRNA-F: 5′-CACCGCAATGAAGAGAGCA CACCTG-3′; sgRNA-R: 5′-AAACCAGGTGTGCTCTCTTCATTG C-3′. HeLa cells were transiently transfected with gRNA-encoding plasmids using TransEasy Transfection Reagent (ForeGene). After 48 h, genome targeting efficiency was primarily determined by T7 endonuclease I (New England Biolabs) digestion, and the cells were then serially diluted into a 96-well plate for the generation of single clones. They were expanded over 2 weeks and screened genotypically for the mutated allele by extracting genomic DNA from subclones, amplifying an approximately 1.0-kb region that encompassed the gRNA-targeted site for sequencing to identify subclones with KO mutations. To validate the occurrence of AAVR KO, we used 50 μg of wild-type or modified HeLa cell lysates for SDS-PAGE separation, and the immunoblots were incubated overnight at 4°C with AAVR antibody (Abcam, ab105385) at a dilution of 1:400. HeLa-WT and HeLa-KO cells were seeded into a 24-well plate at 2×10^5 cells per well 24 h before the experiment. AAV1-, AAV2-, EC71-, and EC73-CMV-LacZ vectors were inoculated onto the cells at MOIs of 10,000, 30,000, and 100,000 in 250 μL of medium. After 1.5 h, the medium was changed, and X-gal staining and β-gal assays were performed 72 h later as described.

SUPPLEMENTAL INFORMATION

Supplemental information can be found online at https://doi.org/10.1016/j.omtm.2021.05.015.

ACKNOWLEDGMENTS

This work was funded by grants from The National Natural Science Foundation of China (31170157 and 81471776) and the National Science and Technology Major Project of the Ministry of Science and Technology of China (2018ZX09201018-013) to L.Y., The National Natural Science Foundation of China (U19A2002) to Y.Y. and L.Y., and the Science and Technology Program of Sichuan Province (20ZDYF2933) to Y.Z. This work was also partially supported by the Research Beyond Borders program from Boehringer Ingelheim. The authors are grateful to Dr. Xiao Xiao of University of North Carolina at Chapel Hill for providing the related plasmids, including UF1-AAV8 and AAV-CMV-luciferase and DNA shuffling plasmid library.

AUTHOR CONTRIBUTIONS

Y.L. conceived and performed experiments, analyzed and interpreted the data, and wrote the manuscript. B.X., Y.C., X.Y., J.L., and T.L. performed experiments. G.D., W.J., Y.Y., Y.Z., L.C., B.D., and Y.W. contributed to conception and design of experiments. L.Y. designed experiments, supervised the project through all stages, and wrote and edited the manuscript.

DECLARATION OF INTERESTS

The authors declare no competing interests.

REFERENCES

1. Melo, L.G., Gnecchi, M., Pachori, A.S., Kong, D., Wang, K., Liu, X., Pratt, R.E., and Dzau, V.J. (2004). Endothelium-targeted gene and cell-based therapies for cardiovascular disease. Arterioscler. Thromb. Vasc. Biol. *24*, 1761–1774.
2. Miller, W.H., Brosnan, M.J., Graham, D., Nicol, C.G., Morecroft, I., Channon, K.M., Danilov, S.M., Reynolds, P.N., Baker, A.H., and Dominiczak, A.F. (2005). Targeting endothelial cells with adenovirus expressing nitric oxide synthase prevents elevation of blood pressure in stroke-prone spontaneously hypertensive rats. Mol. Ther. *12*, 321–327.
3. Wang, X., Cade, R., and Sun, Z. (2005). Human eNOS gene delivery attenuates cold-induced elevation of blood pressure in rats. Am. J. Physiol. Heart Circ. Physiol. *289*, H1161–H1168.
4. Pajusola, K., Gruchala, M., Joch, H., Lüscher, T.F., Ylä-Herttuala, S., and Büeler, H. (2002). Cell-type-specific characteristics modulate the transduction efficiency of adeno-associated virus type 2 and restrain infection of endothelial cells. J. Virol. *76*, 11530–11540.
5. Chen, S., Kapturczak, M., Loiler, S.A., Zolotukhin, S., Glushakova, O.Y., Madsen, K.M., Samulski, R.J., Hauswirth, W.W., Campbell-Thompson, M., Berns, K.I., et al. (2005). Efficient transduction of vascular endothelial cells with recombinant adeno-associated virus serotype 1 and 5 vectors. Hum. Gene Ther. *16*, 235–247.
6. Denby, L., Nicklin, S.A., and Baker, A.H. (2005). Adeno-associated virus (AAV)-7 and -8 poorly transduce vascular endothelial cells and are sensitive to proteasomal degradation. Gene Ther. *12*, 1534–1538.
7. White, S.J., Nicklin, S.A., Büning, H., Brosnan, M.J., Leike, K., Papadakis, E.D., Hallek, M., and Baker, A.H. (2004). Targeted gene delivery to vascular tissue in vivo by tropism-modified adeno-associated virus vectors. Circulation *109*, 513–519.
8. Yang, L., Jiang, J., Drouin, L.M., Agbandje-McKenna, M., Chen, C., Qiao, C., Pu, D., Hu, X., Wang, D.Z., Li, J., and Xiao, X. (2009). A myocardium tropic adeno-associated virus (AAV) evolved by DNA shuffling and in vivo selection. Proc. Natl. Acad. Sci. USA *106*, 3946–3951.
9. Müller, O.J., Kaul, F., Weitzman, M.D., Pasqualini, R., Arap, W., Kleinschmidt, J.A., and Trepel, M. (2003). Random peptide libraries displayed on adeno-associated virus to select for targeted gene therapy vectors. Nat. Biotechnol. *21*, 1040–1046.
10. Körbelin, J., Dogbevia, G., Michelfelder, S., Ridder, D.A., Hunger, A., Wenzel, J., Seismann, H., Lampe, M., Bannach, J., Pasparakis, M., et al. (2016). A brain microvasculature endothelial cell-specific viral vector with the potential to treat neurovascular and neurological diseases. EMBO Mol. Med. *8*, 609–625.
11. Dalkara, D., Byrne, L.C., Klimczak, R.R., Visel, M., Yin, L., Merigan, W.H., Flannery, J.G., and Schaffer, D.V. (2013). In vivo-directed evolution of a new adeno-associated virus for therapeutic outer retinal gene delivery from the vitreous. Sci. Transl. Med. *5*, 189ra76.
12. Lisowski, L., Dane, A.P., Chu, K., Zhang, Y., Cunningham, S.C., Wilson, E.M., Nygaard, S., Grompe, M., Alexander, I.E., and Kay, M.A. (2014). Selection and evaluation of clinically relevant AAV variants in a xenograft liver model. Nature *506*, 382–386.
13. Kisanuki, Y.Y., Hammer, R.E., Miyazaki, J., Williams, S.C., Richardson, J.A., and Yanagisawa, M. (2001). Tie2-Cre transgenic mice: a new model for endothelial cell-lineage analysis in vivo. Dev. Biol. *230*, 230–242.
14. Körbelin, J., Sieber, T., Michelfelder, S., Lunding, L., Spies, E., Hunger, A., Alawi, M., Rapti, K., Indenbirken, D., Müller, O.J., et al. (2016). Pulmonary Targeting of Adeno-associated Viral Vectors by Next-generation Sequencing-guided Screening of Random Capsid Displayed Peptide Libraries. Mol. Ther. *24*, 1050–1061.
15. Morishita, K., Johnson, D.E., and Williams, L.T. (1995). A novel promoter for vascular endothelial growth factor receptor (flt-1) that confers endothelial-specific gene expression. J. Biol. Chem. *270*, 27948–27953.
16. Minami, T., Donovan, D.J., Tsai, J.C., Rosenberg, R.D., and Aird, W.C. (2002). Differential regulation of the von Willebrand factor and Flt-1 promoters in the endothelium of hypoxanthine phosphoribosyltransferase-targeted mice. Blood *100*, 4019–4025.

17. Katusic, Z.S., Caplice, N.M., and Nath, K.A. (2003). Nitric oxide synthase gene transfer as a tool to study biology of endothelial cells. Arterioscler. Thromb. Vasc. Biol. *23*, 1990–1994.

18. Scherrer-Crosbie, M., Ullrich, R., Bloch, K.D., Nakajima, H., Nasseri, B., Aretz, H.T., Lindsey, M.L., Vançon, A.C., Huang, P.L., Lee, R.T., et al. (2001). Endothelial nitric oxide synthase limits left ventricular remodeling after myocardial infarction in mice. Circulation *104*, 1286–1291.

19. Jones, S.P., Greer, J.J., van Haperen, R., Duncker, D.J., de Crom, R., and Lefer, D.J. (2003). Endothelial nitric oxide synthase overexpression attenuates congestive heart failure in mice. Proc. Natl. Acad. Sci. USA *100*, 4891–4896.

20. Ozaki, M., Kawashima, S., Yamashita, T., Hirase, T., Namiki, M., Inoue, N., Hirata, K., Yasui, H., Sakurai, H., Yoshida, Y., et al. (2002). Overexpression of endothelial nitric oxide synthase accelerates atherosclerotic lesion formation in apoE-deficient mice. J. Clin. Invest. *110*, 331–340.

21. van Haperen, R., de Waard, M., van Deel, E., Mees, B., Kutryk, M., van Aken, T., Hamming, J., Grosveld, F., Duncker, D.J., and de Crom, R. (2002). Reduction of blood pressure, plasma cholesterol, and atherosclerosis by elevated endothelial nitric oxide. J. Biol. Chem. *277*, 48803–48807.

22. Opie, S.R., Warrington, K.H., Jr., Agbandje-McKenna, M., Zolotukhin, S., and Muzyczka, N. (2003). Identification of amino acid residues in the capsid proteins of adeno-associated virus type 2 that contribute to heparan sulfate proteoglycan binding. J. Virol. *77*, 6995–7006.

23. Pillay, S., Meyer, N.L., Puschnik, A.S., Davulcu, O., Diep, J., Ishikawa, Y., Jae, L.T., Wosen, J.E., Nagamine, C.M., Chapman, M.S., and Carette, J.E. (2016). An essential receptor for adeno-associated virus infection. Nature *530*, 108–112.

24. Lin, K.F., Chao, L., and Chao, J. (1997). Prolonged reduction of high blood pressure with human nitric oxide synthase gene delivery. Hypertension *30*, 307–313.

25. Hobson, B., and Denekamp, J. (1984). Endothelial proliferation in tumours and normal tissues: continuous labelling studies. Br. J. Cancer *49*, 405–413.

26. Schulick, A.H., Dong, G., Newman, K.D., Virmani, R., and Dichek, D.A. (1995). Endothelium-specific in vivo gene transfer. Circ. Res. *77*, 475–485.

27. Cabanes-Creus, M., Ginn, S.L., Amaya, A.K., Liao, S.H.Y., Westhaus, A., Hallwirth, C.V., Wilmott, P., Ward, J., Dilworth, K.L., Santilli, G., et al. (2018). Codon-Optimization of Wild-Type Adeno-Associated Virus Capsid Sequences Enhances DNA Family Shuffling while Conserving Functionality. Mol. Ther. Methods Clin. Dev. *12*, 71–84.

28. Ojala, D.S., Sun, S., Santiago-Ortiz, J.L., Shapiro, M.G., Romero, P.A., and Schaffer, D.V. (2018). In Vivo Selection of a Computationally Designed SCHEMA AAV Library Yields a Novel Variant for Infection of Adult Neural Stem Cells in the SVZ. Mol. Ther. *26*, 304–319.

29. Börner, K., Kienle, E., Huang, L.Y., Weinmann, J., Sacher, A., Bayer, P., Stüllein, C., Fakhiri, J., Zimmermann, L., Westhaus, A., et al. (2020). Pre-arrayed Pan-AAV Peptide Display Libraries for Rapid Single-Round Screening. Mol. Ther. *28*, 1016–1032.

30. Byrne, L.C., Day, T.P., Visel, M., Strazzeri, J.A., Fortuny, C., Dalkara, D., Merigan, W.H., Schaffer, D.V., and Flannery, J.G. (2020). In vivo-directed evolution of adeno-associated virus in the primate retina. JCI Insight *5*, e135112.

31. Nonnenmacher, M., and Weber, T. (2012). Intracellular transport of recombinant adeno-associated virus vectors. Gene Ther. *19*, 649–658.

32. Dudek, A.M., Pillay, S., Puschnik, A.S., Nagamine, C.M., Cheng, F., Qiu, J., Carette, J.E., and Vandenberghe, L.H. (2018). An Alternate Route for Adeno-associated Virus (AAV) Entry Independent of AAV Receptor. J. Virol. *92*, e02213–e02217.

33. Huang, Q., Chan, K.Y., Tobey, I.G., Chan, Y.A., Poterba, T., Boutros, C.L., Balazs, A.B., Daneman, R., Bloom, J.M., Seed, C., and Deverman, B.E. (2019). Delivering genes across the blood-brain barrier: LY6A, a novel cellular receptor for AAV-PHP.B capsids. PLoS ONE *14*, e0225206.

34. Zhang, R., Cao, L., Cui, M., Sun, Z., Hu, M., Zhang, R., Stuart, W., Zhao, X., Yang, Z., Li, X., et al. (2019). Adeno-associated virus 2 bound to its cellular receptor AAVR. Nat. Microbiol. *4*, 675–682.

35. Zhang, R., Xu, G., Cao, L., Sun, Z., He, Y., Cui, M., Sun, Y., Li, S., Li, H., Qin, L., et al. (2019). Divergent engagements between adeno-associated viruses with their cellular receptor AAVR. Nat. Commun. *10*, 3760–3770.

36. Xiao, X., Li, J., and Samulski, R.J. (1998). Production of high-titer recombinant adeno-associated virus vectors in the absence of helper adenovirus. J. Virol. *72*, 2224–2232.

37. Baudin, B., Bruneel, A., Bosselut, N., and Vaubourdolle, M. (2007). A protocol for isolation and culture of human umbilical vein endothelial cells. Nat. Protoc. *2*, 481–485.

38. Chen, S.F., Fei, X., and Li, S.H. (1995). A new simple method for isolation of microvascular endothelial cells avoiding both chemical and mechanical injuries. Microvasc. Res. *50*, 119–128.

39. Alphonse, R.S., Vadivel, A., Zhong, S., McConaghy, S., Ohls, R., Yoder, M.C., and Thébaud, B. (2015). The isolation and culture of endothelial colony-forming cells from human and rat lungs. Nat. Protoc. *10*, 1697–1708.

40. Zhang, R., Min, W., and Sessa, W.C. (1995). Functional analysis of the human endothelial nitric oxide synthase promoter. Sp1 and GATA factors are necessary for basal transcription in endothelial cells. J. Biol. Chem. *270*, 15320–15326.

41. Leach, J.P., Heallen, T., Zhang, M., Rahmani, M., Morikawa, Y., Hill, M.C., Segura, A., Willerson, J.T., and Martin, J.F. (2017). Hippo pathway deficiency reverses systolic heart failure after infarction. Nature *550*, 260–264.

42. Pacher, P., Nagayama, T., Mukhopadhyay, P., Bátkai, S., and Kass, D.A. (2008). Measurement of cardiac function using pressure-volume conductance catheter technique in mice and rats. Nat. Protoc. *3*, 1422–1434.

43. Wu, Z., Asokan, A., Grieger, J.C., Govindasamy, L., Agbandje-McKenna, M., and Samulski, R.J. (2006). Single amino acid changes can influence titer, heparin binding, and tissue tropism in different adeno-associated virus serotypes. J. Virol. *80*, 11393–11397.

OMTM, Volume *22*

Supplemental information

Directed evolution of AAV accounting for long-term and enhanced transduction of cardiovascular endothelial cells *in vivo*

Y.B. Liu, B.C. Xu, Y.T. Chen, X. Yuan, J.Y. Liu, T. Liu, G.Z. Du, W. Jiang, Y. Yang, Y. Zhu, L.J. Chen, B.S. Ding, Y.Q. Wei, and L. Yang

MATERIALS AND METHODS

Co-localization of AAV-mediated transgene expression and cardiac endothelial cells

3×10^{11} vector genomes of EC71-CMV-GFP vector was injected into male 7-week-old C57BL/6J mice via tail vein. After two weeks, the heart was collected and fixed with 4% paraformaldehyde at 4°C for 12 hours, and then transferred to 30% sucrose for dehydration at 4°C for 24 hours. Tissue for immunostaining was embedded in OCT and 9 µm frozen sections were prepared. Sections were fixed with 4% paraformaldehyde at room temperature for 5 minutes and incubated overnight at 4°C with the rat anti-CD31 (BD Pharmingen, 557355) and chicken anti-GFP (Abcam, ab13970) primary antibodies at a 1:200 dilution in blocking buffer (1% BSA, 10% donkey serum and 0.025% Tween 20 in PBS). Sections were later protected from light and incubated for one hour at room temperature with 1% BSA and 5% donkey serum in PBS containing both donkey anti-rat IgG antibodies conjugated with Alexa Fluor 647 (Jackson, 712-605-150) and donkey anti-chicken IgG, Alexa Fluor 488 (Jackson, 703-545-155) secondary antibodies at a 1:500 dilution. The images were visualized with a confocal microscope (Zeiss) and analyzed with ZEN software. Images were not manipulated in any way except to make brightness and contrast adjustments.

Characterization of Flt1 and eNOS promoter

The AAV-eNOS-luc plasmid was constructed by replacing the CMV promoter in AAV-CMV-luc plasmid with the eNOS promoter[1] (-1033/+22) from the pGL2 enhancer-F1 LUC plasmid (Addgene). EC71-Flt1-luc and EC71-eNOS-luc vectors were produced using the three-plasmid co-transfection method and CsCl gradient density centrifugation. 3×10^{11} or 1×10^{12} vector genomes of these vectors were injected into the male 7-week-old C57BL/6J mice via tail vein. Three weeks later, mouse heart, liver, brain, and lung tissues were harvested for luciferase assay as previously described.

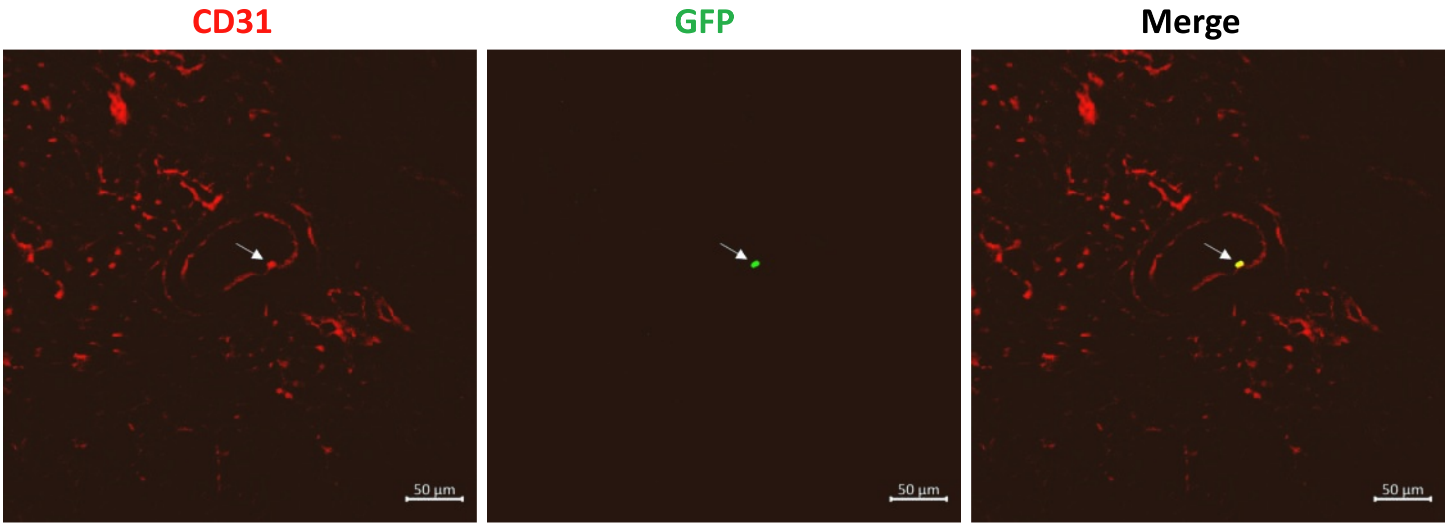

Figure S1. EC71 transduced cardiac endothelial cells in vivo. $3x10^{11}$ vector genomes of EC71-CMV-GFP were injected into the young adult C57B6 mice intravenously and transgene expression within mouse heart was detected by immunostaining two weeks later. The representative images for GFP transgene expression (green) in the cardiac endothelial cells co-stained with CD31 marker (red) were demonstrated. The white arrows showed co-staining.

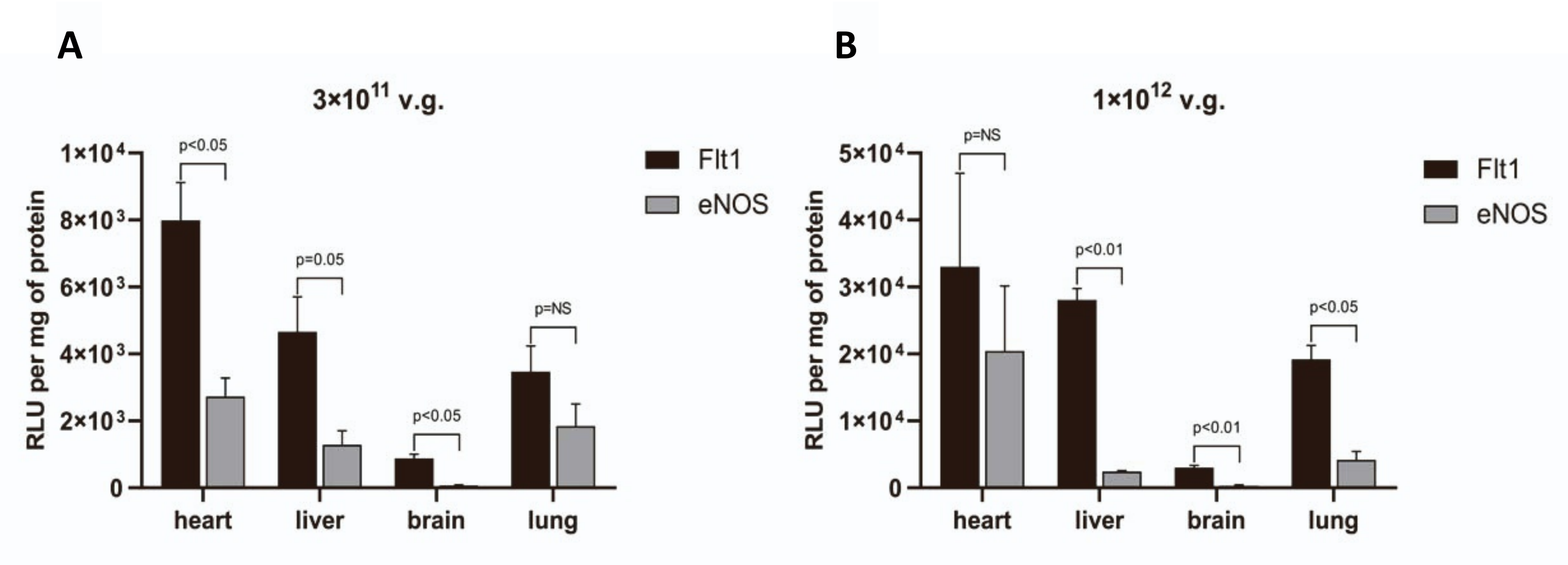

Figure S2. Characterization of the endothelial-specific Flt-1 and eNOS promoters. 3×10^{11} (A) or 1×10^{12} (B) vector genomes of EC71 vector encompassing luciferase gene were injected intravenously into adult C57B6 mice. Three weeks later, luciferase activities among mouse tissues were measured to compare these two promoters on their strength and tissue specificity. Data are mean values ± SD.

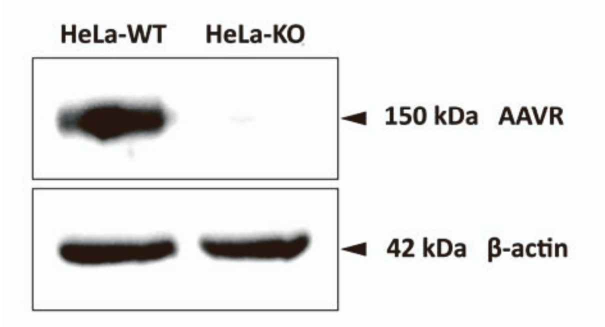

Figure S3. AAVR expression in HeLa cell strains. HeLa-WT and HeLa-KO cell lysates were separated by SDS-PAGE and immunoblotted with a commercial AAVR antibody. One approximate 150 kDa band correspondent to glycosylated AAVR was observed for the HeLa-WT cells but lacked for HeLa-KO cells.

REFERENCES

1. Zhang, R., Min, W., and Sessa, W. C. (1995). Functional analysis of the human endothelial nitric oxide synthase promoter. Sp1 and GATA factors are necessary for basal transcription in endothelial cells. J. Biol. Chem. 270, 15320-15326.
